# 1-Methyl-1*H*-benzimidazole-2(3*H*)-thione

**DOI:** 10.1107/S1600536808015043

**Published:** 2008-05-24

**Authors:** Hizbullah Khan, Amin Badshah, Farkhanda Shaheen, Christine Gieck, Rizwana Aleem Qureshi

**Affiliations:** a Department of Chemistry, Quaid-i-Azam University, Islamabad, Pakistan; b DISTA, Universita del Piemonte Orientale, Alessandria I-15100, Italy; c Department of Plant Sciences, Faculty of Biological Sciences, Quaid-I-Azam University, Islamabad 45320, Pakistan

## Abstract

The title compound, C_8_H_8_N_2_S, was prepared by the condensation of *N*-methyl-1,2-phenyl­enediamine and carbon disulfide. The crystal structure is stabilized by a C—H⋯π inter­action between a benzene H atom and the benzene ring of a neighbouring mol­ecule, and by inter­molecular N—H⋯S inter­actions.

## Related literature

For related literature, see: Baily *et al.* (1996 ▶); Koch (2001 ▶); Namgun *et al.* (2001 ▶); Schuster *et al.* (1990 ▶); Patel & Chedekel (1984 ▶).
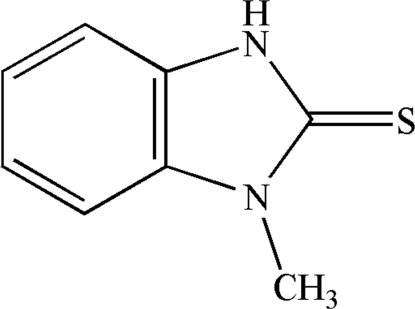


## Experimental

### 

#### Crystal data

C_8_H_8_N_2_S
*M* *_r_* = 164.22
Monoclinic, 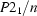

*a* = 9.997 (4) Å
*b* = 5.8140 (7) Å
*c* = 13.703 (4) Å
β = 94.05 (3)°
*V* = 794.5 (4) Å^3^
*Z* = 4
Mo *K*α radiation
μ = 0.34 mm^−1^
*T* = 293 (2) K
0.20 × 0.10 × 0.02 mm

#### Data collection

Oxford Diffraction Xcalibur2 CCD diffractometer
Absorption correction: analytical (*CrysAlis RED*; Oxford Diffraction; 2004 ▶; Clark & Reid, 1995 ▶) *T*_min_ = 0.929, *T*_max_ = 0.967
7237 measured reflections
962 independent reflections
855 reflections with *I* > 2σ(*I*)
*R*_int_ = 0.023
θ_max_ = 23.1°

#### Refinement

*R*[*F* ^2^ > 2σ(*F* ^2^)] = 0.029
*wR*(*F* ^2^) = 0.077
*S* = 1.09
962 reflections
101 parameters
H-atom parameters constrained
Δρ_max_ = 0.20 e Å^−3^
Δρ_min_ = −0.19 e Å^−3^

### 

Data collection: *CrysAlis CCD* (Oxford Diffraction, 2004 ▶); cell refinement: *CrysAlis RED* (Oxford Diffraction, 2004 ▶); data reduction: *CrysAlis RED*; program(s) used to solve structure: *SHELXS97* (Sheldrick, 2008 ▶); program(s) used to refine structure: *SHELXL97* (Sheldrick, 2008 ▶); molecular graphics: *PLATON* (Spek, 2003 ▶); software used to prepare material for publication: *PLATON*.

## Supplementary Material

Crystal structure: contains datablocks I, global. DOI: 10.1107/S1600536808015043/lx2055sup1.cif

Structure factors: contains datablocks I. DOI: 10.1107/S1600536808015043/lx2055Isup2.hkl

Additional supplementary materials:  crystallographic information; 3D view; checkCIF report

## Figures and Tables

**Table 1:** Hydrogen-bond geometry (Å, °)

*D*—H⋯*A*	*D*—H	H⋯*A*	*D*⋯*A*	*D*—H⋯*A*
N2—H2⋯S^i^	0.86	2.57	3.408 (2)	166
C3—H3⋯*Cg*^ii^	0.93	2.74	3.464 (3)	136

Symmetry codes: (i) 
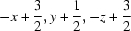
; (ii) 
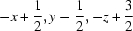
. *Cg* is the centroid of the C2–C7 ring.

**Fig. 1.**
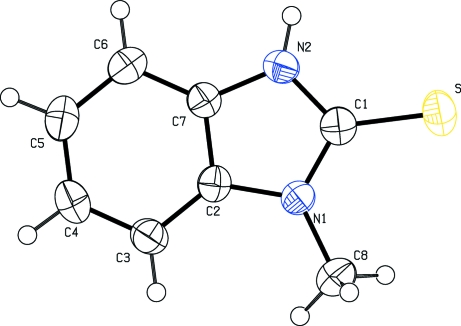
The structure of (I), with displacement ellipsoids drawn at the 50% probability level.

**Fig. 2.**
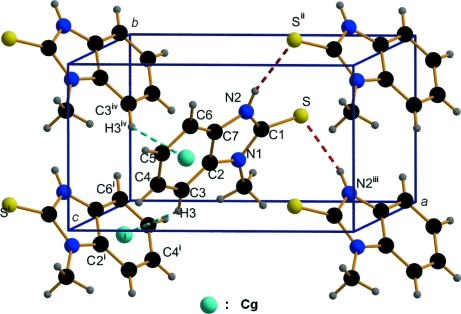
C—H···π and N—H···S interactions (dotted lines) in the title compound. Cg denotes the ring centroid. [Symmetry code: (i) -x+1/2, y-1/2, -z+3/2; (ii) -x+3/2, y+1/2, -z+3/2; (iii) -x+3/2, y-1/2, -z+3/2; (iv) -x+1/2, y+1/2, -z+3/2.]

## supplementary crystallographic information

### Comment

N,*N*'- disubstituted and N-substituted thiourea derivatives are the major building blocks of organic macromolecular compounds. Thiourea derivatives such as benzothiazoles have been isolated by bromination of arylthioureas (Patil & Chedekel, 1984) and by condensation of 2-aminothiazole (Baily *et al.*, 1996), by cyclization of *N*-(2-hydroxyethyl-*N*-methylthioureas and 2-methyl-aminothiazole (Namgun *et al.*, 2001). Aliphatic and acylthioureas have a wide range of application due to their coordination behavior towards transition metals (Schuster *et al.*, 1990). *N*,*N*-dialkyl-*N*-arylthioureas have been used for the extraction of metals such as nickel, palladium and platinum (Koch, 2001). Here we report the crystal structure of the title compound, 1-methyl-2H-benzimidazole-2-thione (Fig. 1).

The benzimidazole unit is essentially planar, with a mean deviation of 0.023 Å from the least-squares plane defined by the nine constituent atoms. The molecular packing (Fig. 2) is stabilized by a C—H···π interaction between a benzene H atom and the benzene ring of neighbouring molecules, with a C3—H3···Cg^i^ separation of 2.735 (3) Å (Fig. 2 and Table 1; Cg is the C2-C7 benzene ring, symmetry code as in Fig. 2). Additionally, intermolecular N—H···S interactions in the structure were observed (Fig. 2 and Table 1; symmetry code as in Fig. 2).

### Experimental

Compound (I) was synthesized by the addition of carbondisulfide (0.79 ml, 13.02 mmol) to *N*-methyl-1,2-phenylenediamine (0.744 ml, 6.55 mmol) in methanol (20 ml). The resulting mixture was stirred for 24 h, at 0°C temperature, giving a clear light yellow solution. The solution was evaporated under reduced pressure to give a light yellow solid, which was recrystallized in methanol/peteroleum ether (9:1) to afford compound (I) (yield : 76%).

### Refinement

All H atoms were placed in idealized positions (C—H = 0.96 A ° (methyl); C—H = 0.93 A ° (aromatic); N—H = 0.86 A °) and refined as riding, with *U*_iso_(H) = 1.2*U*_eq_(C, N) or 1.5*U*_eq_(C).

### Crystal data

**Table:**

C_8_H_8_N_2_S	*F*_000_ = 344
*M**_r_* = 164.22	*D*_x_ = 1.373 Mg m^−^^3^
Monoclinic, *P*2_1_/*n*	Melting point: 402 K
Hall symbol: -P_2yn	Mo *K*α radiation λ = 0.71073 Å
*a* = 9.997 (4) Å	Cell parameters from 5701 reflections
*b* = 5.8140 (7) Å	θ = 3.7–23.1º
*c* = 13.703 (4) Å	µ = 0.34 mm^−^^1^
β = 94.05 (3)º	*T* = 293 (2) K
*V* = 794.5 (4) Å^3^	Block, colourless
*Z* = 4	0.20 × 0.10 × 0.02 mm

### Data collection

**Table:**

Oxford Diffraction Xcalibur2 CCD diffractometer	962 independent reflections
Radiation source: Enhance (Mo) X-ray Source	855 reflections with *I* > 2σ(*I*)
Monochromator: graphite	*R*_int_ = 0.023
Detector resolution: 10.0 pixels mm^-1^	θ_max_ = 23.1º
*T* = 293(2) K	θ_min_ = 3.8º
ω scans	*h* = −10→11
Absorption correction: analytical(CrysAlis RED; Oxford Diffraction; 2004; Clark & Reid, 1995)	*k* = −6→6
*T*_min_ = 0.929, *T*_max_ = 0.967	*l* = −14→14
7237 measured reflections	

### Refinement

**Table:**

Refinement on *F*^2^	Secondary atom site location: difference Fourier map
Least-squares matrix: full	Hydrogen site location: inferred from neighbouring sites
*R*[*F*^2^ > 2σ(*F*^2^)] = 0.029	H-atom parameters constrained
*wR*(*F*^2^) = 0.078	*w* = 1/[σ^2^(*F*_o_^2^) + (0.0353*P*)^2^ + 0.4983*P*] where *P* = (*F*_o_^2^ + 2*F*_c_^2^)/3
*S* = 1.09	(Δ/σ)_max_ = 0.001
962 reflections	Δρ_max_ = 0.21 e Å^−^^3^
101 parameters	Δρ_min_ = −0.19 e Å^−^^3^
Primary atom site location: structure-invariant direct methods	Extinction correction: none

### Special details

**Table:**

Geometry. All esds (except the esd in the dihedral angle between two l.s. planes) are estimated using the full covariance matrix. The cell esds are taken into account individually in the estimation of esds in distances, angles and torsion angles; correlations between esds in cell parameters are only used when they are defined by crystal symmetry. An approximate (isotropic) treatment of cell esds is used for estimating esds involving l.s. planes.
Refinement. Refinement of F^2^ against ALL reflections. The weighted R-factor wR and goodness of fit S are based on F^2^, conventional R-factors R are based on F, with F set to zero for negative F^2^. The threshold expression of F^2^ > 2sigma(F^2^) is used only for calculating R-factors(gt) etc. and is not relevant to the choice of reflections for refinement. R-factors based on F^2^ are statistically about twice as large as those based on F, and R- factors based on ALL data will be even larger.

### Fractional atomic coordinates and isotropic or equivalent isotropic displacement parameters (Å^2^)

**Table:**

	*x*	*y*	*z*	*U*_iso_*/*U*_eq_	
S	0.73449 (6)	0.62833 (10)	0.62836 (4)	0.0475 (3)	
N1	0.52959 (17)	0.3736 (3)	0.69273 (12)	0.0345 (5)	
N2	0.57646 (17)	0.6789 (3)	0.78071 (12)	0.0380 (5)	
H2	0.6119	0.8071	0.8004	0.046*	
C1	0.6119 (2)	0.5599 (4)	0.70145 (15)	0.0359 (6)	
C2	0.4462 (2)	0.3690 (3)	0.77058 (15)	0.0323 (5)	
C3	0.3528 (2)	0.2095 (4)	0.79703 (16)	0.0399 (6)	
H3	0.3342	0.0773	0.7603	0.048*	
C4	0.2882 (2)	0.2566 (4)	0.88107 (17)	0.0461 (6)	
H4	0.2255	0.1524	0.9017	0.055*	
C5	0.3145 (2)	0.4548 (4)	0.93499 (17)	0.0466 (6)	
H5	0.2684	0.4812	0.9906	0.056*	
C6	0.4079 (2)	0.6150 (4)	0.90824 (16)	0.0412 (6)	
H6	0.4246	0.7492	0.9440	0.049*	
C7	0.4752 (2)	0.5656 (4)	0.82572 (15)	0.0330 (5)	
C8	0.5331 (3)	0.1997 (4)	0.61682 (17)	0.0518 (7)	
H8A	0.5508	0.2717	0.5560	0.078*	
H8B	0.4482	0.1221	0.6098	0.078*	
H8C	0.6026	0.0904	0.6345	0.078*	

### Atomic displacement parameters (Å^2^)

**Table:**

	*U*^11^	*U*^22^	*U*^33^	*U*^12^	*U*^13^	*U*^23^
S	0.0446 (4)	0.0454 (4)	0.0547 (4)	0.0026 (3)	0.0184 (3)	0.0102 (3)
N1	0.0345 (10)	0.0313 (10)	0.0384 (10)	0.0019 (8)	0.0071 (8)	−0.0027 (8)
N2	0.0379 (11)	0.0323 (10)	0.0443 (11)	−0.0042 (8)	0.0064 (9)	−0.0040 (9)
C1	0.0350 (12)	0.0332 (12)	0.0396 (13)	0.0068 (11)	0.0026 (10)	0.0050 (10)
C2	0.0283 (11)	0.0339 (13)	0.0348 (12)	0.0062 (10)	0.0024 (9)	0.0023 (10)
C3	0.0365 (12)	0.0356 (13)	0.0478 (14)	−0.0024 (11)	0.0043 (11)	−0.0016 (11)
C4	0.0380 (13)	0.0483 (16)	0.0529 (15)	−0.0053 (11)	0.0094 (12)	0.0080 (13)
C5	0.0425 (13)	0.0601 (16)	0.0383 (13)	0.0015 (13)	0.0104 (11)	0.0021 (12)
C6	0.0423 (13)	0.0431 (14)	0.0380 (13)	0.0040 (11)	0.0017 (10)	−0.0060 (11)
C7	0.0302 (12)	0.0330 (12)	0.0358 (12)	0.0021 (10)	0.0019 (10)	0.0020 (10)
C8	0.0536 (15)	0.0493 (15)	0.0542 (15)	−0.0015 (13)	0.0150 (12)	−0.0150 (13)

### Geometric parameters (Å, °)

**Table:**

S—C1	1.684 (2)	C3—H3	0.9300
N1—C1	1.361 (3)	C4—C5	1.384 (3)
N1—C2	1.400 (3)	C4—H4	0.9300
N1—C8	1.453 (3)	C5—C6	1.386 (3)
N2—C1	1.356 (3)	C5—H5	0.9300
N2—C7	1.389 (3)	C6—C7	1.386 (3)
N2—H2	0.8600	C6—H6	0.9300
C2—C3	1.383 (3)	C8—H8A	0.9600
C2—C7	1.389 (3)	C8—H8B	0.9600
C3—C4	1.387 (3)	C8—H8C	0.9600
			
C1—N1—C2	109.71 (17)	C3—C4—H4	119.2
C1—N1—C8	124.88 (18)	C4—C5—C6	121.6 (2)
C2—N1—C8	125.34 (18)	C4—C5—H5	119.2
C1—N2—C7	110.71 (18)	C6—C5—H5	119.2
C1—N2—H2	124.6	C7—C6—C5	116.8 (2)
C7—N2—H2	124.6	C7—C6—H6	121.6
N2—C1—N1	106.62 (18)	C5—C6—H6	121.6
N2—C1—S	126.72 (18)	C6—C7—N2	132.5 (2)
N1—C1—S	126.65 (17)	C6—C7—C2	121.3 (2)
C3—C2—C7	121.86 (19)	N2—C7—C2	106.21 (17)
C3—C2—N1	131.46 (19)	N1—C8—H8A	109.5
C7—C2—N1	106.66 (17)	N1—C8—H8B	109.5
C2—C3—C4	116.6 (2)	H8A—C8—H8B	109.5
C2—C3—H3	121.7	N1—C8—H8C	109.5
C4—C3—H3	121.7	H8A—C8—H8C	109.5
C5—C4—C3	121.7 (2)	H8B—C8—H8C	109.5
C5—C4—H4	119.2		
			
C7—N2—C1—N1	2.4 (2)	C2—C3—C4—C5	0.9 (3)
C7—N2—C1—S	−177.28 (16)	C3—C4—C5—C6	−0.7 (4)
C2—N1—C1—N2	−3.2 (2)	C4—C5—C6—C7	−1.0 (3)
C8—N1—C1—N2	179.79 (19)	C5—C6—C7—N2	−177.1 (2)
C2—N1—C1—S	176.50 (15)	C5—C6—C7—C2	2.4 (3)
C8—N1—C1—S	−0.5 (3)	C1—N2—C7—C6	178.9 (2)
C1—N1—C2—C3	−175.6 (2)	C1—N2—C7—C2	−0.7 (2)
C8—N1—C2—C3	1.4 (3)	C3—C2—C7—C6	−2.3 (3)
C1—N1—C2—C7	2.8 (2)	N1—C2—C7—C6	179.11 (19)
C8—N1—C2—C7	179.79 (19)	C3—C2—C7—N2	177.32 (19)
C7—C2—C3—C4	0.6 (3)	N1—C2—C7—N2	−1.2 (2)
N1—C2—C3—C4	178.8 (2)		

### Hydrogen-bond geometry (Å, °)

**Table:**

*D*—H···*A*	*D*—H	H···*A*	*D*···*A*	*D*—H···*A*
N2—H2···S^i^	0.86	2.57	3.408 (2)	166
C3—H3···Cg^ii^	0.93	2.74	3.464 (3)	136

Symmetry codes: (i) −*x*+3/2, *y*+1/2, −*z*+3/2; (ii) −*x*+1/2, *y*−1/2, −*z*+3/2.
